# Fracture Healing in Patients With HIV in South Africa: A Prospective Cohort Study

**DOI:** 10.1097/QAI.0000000000002720

**Published:** 2021-05-10

**Authors:** Simon M. Graham, Sithombo Maqungo, Maritz Laubscher, Nando Ferreira, Michael Held, William J. Harrison, A. Hamish Simpson, Peter MacPherson, David G. Lalloo

**Affiliations:** a Institute of Population Health Data Science, University of Liverpool, Liverpool, United Kingdom;; b Department of Orthopaedic and Trauma Surgery, Liverpool University Teaching Hospital Trust, Liverpool, United Kingdom;; c Division of Orthopaedic Surgery, Groote Schuur Hospital, Cape Town, South Africa;; d Division of Global Surgery, Orthopaedic Research Unit (ORU), University of Cape Town, Cape Town, South Africa;; e Division of Orthopaedic Surgery, Stellenbosch University, Cape Town, South Africa;; f Department of Orthopaedic and Trauma Surgery, Countess of Chester Hospital, Chester, United Kingdom;; g University of Edinburgh, Edinburgh, United Kingdom;; h Malawi-Liverpool-Wellcome Trust Clinical Research Programme, Blantyre, Malawi;; i Clinical Research Department, London School of Hygiene and Tropical Medicine, London, United Kingdom; and; j Department of Clinical Sciences, Liverpool School of Tropical Medicine, Liverpool, United Kingdom.

**Keywords:** bone healing, delayed union, fracture, human immunodeficiency virus, intramedullary nailing, nonunion, union

## Abstract

HIV reduces bone mineral density, mineralization, and turnover and may impair fracture healing.

**Setting:**

This prospective cohort study in South Africa investigated whether HIV infection was associated with impaired fracture healing after trauma.

**Methods:**

All adults with acute tibia and femur fractures who underwent intermedullary (IM) nailing for fracture fixation between September 2017 and December 2018, at 2 tertiary hospitals, were followed up for a minimum of 12 months postoperatively. The primary outcome was delayed bone union at 6 months (defined by the radiological union scoring system for the tibia score <9), and the secondary outcome was nonunion (defined as radiological union scoring system for the tibia score <9) at 9 months. Multivariable logistic regression models were constructed to investigate the associations between HIV status and impaired fracture healing.

**Results:**

In total, 358 participants, who underwent 395 IM nailings, were enrolled in the study and followed up for 12 months. Seventy-one of the 358 (19.8%) participants were HIV-positive [83/395 (21%) IM nailings]. HIV was not associated with delayed fracture healing after IM nailing of the tibia or femur (multivariable odds ratio: 1.06; 95% confidence interval: 0.50 to 2.22). HIV-positive participants had a statistically significant lower odds ratio of nonunion compared with HIV-negative participants (multivariable odds ratio: 0.17; 95% confidence interval: 0.01 to 0.92).

**Conclusions:**

Fractures sustained in HIV-positive individuals can undergo surgical fixation as effectively as those in HIV-negative individuals, with no increased risk of delayed union or nonunion.

## Abstract 2

Supplemental Digital Content is Available in the Text.

## INTRODUCTION

Worldwide, approximately 38 million people are living with HIV infection.^1^ Ninety-one percentage of the people living with HIV reside in a low-income or middle-income country,^2^ with an estimated 68% living in sub-Saharan Africa.^1^ Widespread availability of antiretroviral therapy (ART) in sub-Saharan Africa since 2002 has altered the course and nature of the HIV epidemic, with HIV-positive individuals attaining healthy, close-to-normal life spans.^3^

Similar to the distribution of the HIV burden, it is estimated that 83% of injury-related deaths worldwide occur in low-income or middle-income countries, accounting for more deaths than those from malaria, tuberculosis, and HIV combined.^4^ For every injury-related death, it is estimated that 10–50 people sustain permanent or temporary disabilities: musculoskeletal trauma accounts for most of these injuries.^5^ It is, therefore, likely that a considerable proportion of people presenting with musculoskeletal injuries in sub-Saharan Africa are HIV-positive. However, the effects of the long-term immunosuppression resulting from HIV infection on the fracture-repair process after a musculoskeletal injury are not well understood.

A number of factors could theoretically affect fracture healing. HIV principally affects immunological status by exhausting the host CD4 T cells, resulting in an increase in the risk of opportunistic infections. HIV also affects other cellular chemical mediators, including interleukins 1 and 6 (IL1, IL6), and tumor necrosis factor, which have been shown to play a role in the fracture-repair process.^6,7^ HIV-associated and ART-associated interruption in osseous blood supply can also lead to osteonecrosis,^8,9^ and this microvascular effect may lead to higher rates of delayed fracture healing and nonunion.^10^ Reduced bone mineral density, mineralization, and turnover have also been shown to occur in HIV-positive individuals and those on ART.^11,12^ This not only increases the risk of fragility fractures but could also potentially influence fracture healing.^13^

A small number of clinical studies have investigated the role of HIV in the fracture-healing process.^14–16^ These studies suggest that HIV and/or ART are associated with delayed fracture healing and may result in nonunion, although patient numbers were small, and no underlying mechanisms were identified. If this hypothesis was shown to be true, the surgical management of fractures could be tailored to optimize bone union during the fracture-healing phase in HIV-positive patients, improving outcomes and reducing the substantial physical and social burden that occurs in these patients because of traumatic injuries.

Overall, the true effects of HIV and ART on bone healing are very poorly understood. This article reports the findings of the HIV in Orthopedic Skeletal Trauma (HOST) study, which aimed to investigate whether HIV infection is a risk factor for the development of delayed union or nonunion after a fracture.

## METHODS

### Study Design and Participants

The HOST study was a multicenter prospective study of patients undergoing fracture surgery at 2 tertiary referral hospitals—Groote Schuur Hospital and Tygerberg Hospital—in Cape Town, South Africa. Recruitment was undertaken over a 14-month period, between September 2017 and December 2018.

Patients were eligible for inclusion if they were 18 years or older at assessment and had sustained a closed or open fracture of the shaft of the tibia or femur, which was treated with intramedullary (IM) nailing within 2 weeks of injury. Patients were excluded if they had a pathological fracture or a peritrochanteric femur fracture (bone shaft fractures); had a major head injury, had severe burns, or were paraplegic; had presurgical infection at the fracture site or an open injury for >48 hours before the first debridement; or were unwilling to participate in study follow-up protocols, complete questionnaires, or attend follow-up. Participants who had sustained multiple injuries, including tibia or femur fractures, had their injuries documented and were included in the enrollment process.

The HOST study received ethical approval from the ethics committee of the study sites: the University of Cape Town; Faculty of Health Science, the University of Stellenbosch; and the Liverpool School of Tropical Medicine Research. The study protocol has been published earlier.^17^

### Baseline

Participants were recruited postoperatively by one of the 2 research nurses and undertook a baseline questionnaire to record clinical and sociodemographic characteristics, including risk factors of impaired bone healing and nonunion (age, sex, smoking status, nonsteroidal drug use, medical history, vitamin D status, mechanism of injury, open fracture, and injury severity score). Participants not confirmed to be taking ART were offered HIV testing (Alere Determine HIV-1/2 assay; Alere Medical Co. Ltd., Chiba, Japan, and Uni-GoldTM Recombigen; Trinity BioTech, Wicklow, Ireland), with measurement of CD4 cell count (FACScount; Becton Dickinson, BD Biosciences, San Jose, CA) and HIV viral load (bioMe'rieux NucliSENS EasyQ System HIV-1 QT) if they were found to be HIV-positive. Participants newly diagnosed with HIV were linked to HIV care clinics.

All participants were seen in clinic at 6 months postsurgery and were followed up for a minimum of 12 months. Outpatient assessments and x-rays were undertaken at 2 and 6 weeks and at 3, 6, and 9 months to assess bone union. If participants' fracture were confirmed to have united at 6 months, they were followed up by telephone at 9 and 12 months. A nonunion was confirmed if fracture union had not occurred at 9 months after injury. Participants with confirmed nonunions were offered further management, according to local protocols for treatment of nonunion. If, at any time, the responsible consultant surgeon felt that there was a need for further surgery to achieve union before 9 months, it was offered after a joint discussion with at least 2 consultant orthopedic surgeons.

### Definitions and Outcomes

The primary study outcome was the proportion of participants with delayed bone union at 6 months, which was compared between HIV-positive and HIV-negative participants. The secondary study outcomes were nonunion at 9 months and infection.

Bone healing was assessed using the validated radiological union scoring system for tibia (RUST).^18–20^ Fracture union was defined as radiological union on RUST score [score of 3 on at least 3 of the 4 cortices (anterior, lateral, medial, or posterior cortex)—a total RUST score of 9 or more] within 6 months of surgery.^18–20^ Delayed bone union was defined as impaired bone healing at 6 months on RUST score <9.^18–20^ Nonunion was defined as either impaired bone healing at 9 months on RUST score <9^18–20^ or the need for further surgery to achieve union (RUST score <9) before 9 months (decision made by 2 orthopedic surgeons).

Two reviewers (both orthopedic surgeons), blinded to HIV status, independently assessed radiological fracture union on radiographs. In case of discrepancies in RUST scoring between the reviewers, a third reviewer (orthopedic surgeon) independently undertook a review of the radiograph to determine the final outcome.

Infection was diagnosed using the United States Centers for Disease Control and Prevention criteria for “superficial surgical site infection (SSI)” and “deep surgical site infection (DSI).” SSI was defined as a wound infection involving the skin and subcutaneous tissue, which occurred within 30 days of surgery,^21^ and DSI as a wound infection involving the tissues deep to the skin, which occurred within 30 days of injury (closed reduction of fracture) or 90 days (open reduction of fracture).^22^ Late infection was diagnosed as any late-wound breakdown (>30 days for closed reduction of fractures or >90 days for openly reduced fractures) or sinus formation or unexplained late pain with associated radiological changes consistent with periimplant infection.^23^

### Statistical Methods

A previously established orthopedic surgical register suggested that 400 participants were likely to undergo IM nailing of the tibia and femur at the 2 centers over the 14-month study period, and 80% (n = 320) were assumed to be able to complete follow-up to 9 months. On the basis of previous research,^24–26^ it was estimated that 272 of the 320 (85%) participants would have fracture union at 6 months (control), and 48 (15%) would have delayed bone union (cases). Assuming that 20% of participants without delayed union would be HIV-positive, a sample size of 400 participants would give 82.8% power to detect at least a 2-times relative difference in HIV prevalence between participants in case and control groups at the *P* = 0.05 threshold.

Baseline characteristics were summarized using means (with standard deviations), medians [with interquartile ranges (IQRs)], and percentages and compared between HIV-positive and HIV-negative participants. For the primary outcome (delayed union), a multivariable logistic regression model was constructed to estimate the odds ratio (OR) and 95% confidence interval (CI) for delayed union by comparing HIV-positive and HIV-negative participants and adjusting for important confounders, identified a priori through construction of putative causal diagrams. A separate model was constructed for participants with HIV only to estimate the associations between HIV-associated predictors (eg, CD4 cell count, viral load, and ART use) and delayed union. For the secondary outcome (nonunion), a multivariable logistic regression model was constructed to estimate the OR and 95% CI for nonunion by comparing HIV-positive and HIV-negative participants and adjusting for important confounders. Statistical analysis was performed using *R* statistical software.

Some of the enrolled participants had more than one tibia or femur fracture. Therefore, in the analysis, CIs were adjusted for clustering by including a random effects term in regression models. This study was registered with ClinicalTrials.gov (NCT03131947).

## RESULTS

**FIGURE 1.**
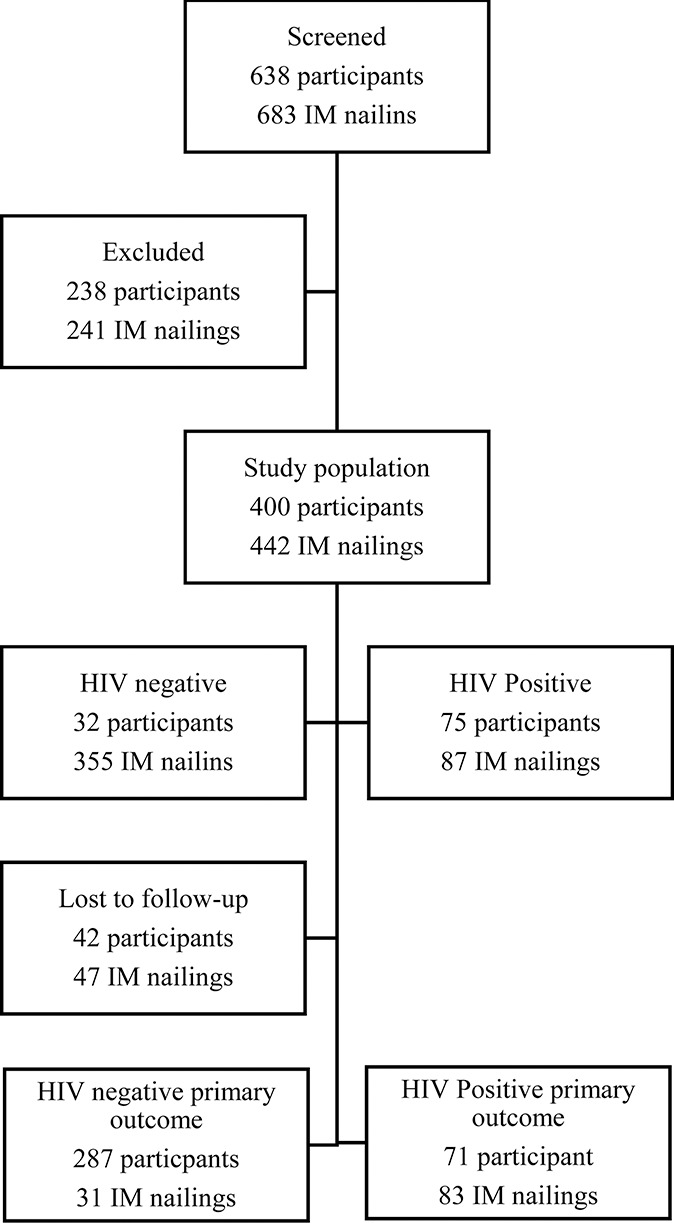
Flow diagram of study population recruitment.

Between September 2017 and December 2018, 638 patients underwent 683 IM nailings of the femur and tibia at the 2 study sites and were screened for study eligibility; 238 participants (241 IM nailings) did not meet the study inclusion criteria, and the remaining 400 participants (442 IM nailings) were enrolled in the study (Fig. 1). Baseline characteristics of all participants are presented in Table 1.

**TABLE 1.** Baseline Characteristics of Study Participants, Stratified by HIV Status

Characteristics	Study Cohort N = 400	HIV-Negative N = 325	HIV-Positive N = 75	*P*
Sex, n (%)				
Men	313 (78.3)	262 (80.6)	51 (68.0)	0.030
Women	87 (21.7)	63 (19.4)	24 (32.0)	
Age, yrs: median (IQR)	32.36 (18–71)	31 (18–71)	35 (19–58)	0.080
BMI, kg/m^2^: median (IQR)	23.02 (15.54–51.19)	22.9 (15.72–47.5)	23.31 (15.55–51.2)	0.720
Fracture site,* n (%)				
Tibia	215 (48.6)	171 (48.2)	44 (50.6)	0.460
Femur	227 (51.4)	184 (51.8)	43 (49.4)	
Open fracture,* n (%)				
Yes	161 (36.4)	139 (39.2)	22 (25.3)	0.059
No	281 (63.6)	216 (60.8)	65 (74.7)	
No. of IM nailings performed per participant, n (%)				
1 nail	361 (90.3)	296 (91.1)	65 (86.7)	0.340
2 nails	37 (9.3)	28 (8.6)	9 (12.0)	
3 nails	1 (0.2)	1 (0.3)	0	
4 nails	1 (0.2)	0	1 (1.3)	
Drinks any alcohol, n (%)				
Yes	223 (55.8)	181 (55.7)	42 (56.0)	0.940
No	177 (44.2)	144 (44.3)	33 (44.0)	
Smoking status, n (%)				
Nonsmoker	175 (43.8)	134 (41.2)	41 (54.7)	0.050
Smoker	225 (56.2)	191 (58.8)	34 (45.3)	
Transfer from district hospital, n (%)				
Yes	98 (24.5)	74 (22.8)	24 (32.0)	
No	302 (75.5)	251 (77.2)	51 (68.0)	0.100
Time taken to arrive at treating hospital, hrs: median (IQR)	9 (4–24)	10 (4–24)	12 (6–18)	0.840
Patient-reported outcome measure (PROM)				
DRI preoperatively, median (IQR)	0 (0–34.3)	0 (0–28)	0 (0–34.3)	0.090

* N = 442 for study cohort, 355 HIV-negative, 87 HIV-positive.

BMI, body mass index; DRI, disability rating index.

The overall prevalence of HIV in the study population was 18.8% (75/400 participants). The 75 HIV participants with HIV underwent 87 IM nailings, giving an overall prevalence of HIV per IM nail of 19.7% (87/442). Just more than half of participants with HIV (42/75, 56.0%) knew the results of their HIV diagnosis before enrollment in the study. The median length of time a participant had had a diagnosis of HIV was 1397 days (IQR 686–3565 days) or 3.8 years (IQR 1.9–9.8 years). The remaining participants were diagnosed during their admission (25/75, 33.3%) or within 2 weeks of their discharge (8/75, 10.7%). Baseline characteristics of participants, stratified by HIV status, are summarized in Table 2.

**TABLE 2.** Baseline Characteristics of HIV-Positive Participants

HIV Status (N = 400)	
HIV-positive	75 (18.8)
HIV-negative	325 (81.2)

HIV Parameter (N = 75)	
Diagnosis, n (%)	
Before admission	42 (56.0)
On or during admission	25 (33.3)
After discharge	8 (10.7)
Age at time of HIV diagnosis, yrs: median (IQR)	32.36 (17.46–48.23)
Length of time with HIV diagnosis,* d: median (IQR)	1397 (686–3565)
Taking ART on admission, n (%)	
Yes	42 (56.0)
No	33 (44.0)
Length of time taking ART therapy, d: median (IQR)	1732 (678–3568)
ART (N = 42), n (%)	
TDF, 3TC + EFV	38 (90.5)
TDF, FTC + EFV	1 (2.4)
ZDV, 3TC + LPV/r	3 (7.1)
CD4^+^ count on admission, cells/mm^3^: median (IQR)	393 (63–1145)
Viral load (copies/mL) on admission, log_10_ copies/mL: median (IQR)	2.13 (1.3–4.62)

* Participants with a previous diagnosis of HIV before admission.

3TC, lamivudine; ; EFV, efavirenz; FTC, emtricitabine; LPV/r, lopinavir/ritonavir; TDF, tenofovir; ZDV, zidovudine.

All 442 fractures underwent reamed locked (proximally and distally) IM nailings across the 2 study sites. The procedures were undertaken predominantly by registrar or equivalent training-level surgeons (98.4%, 435/442). A total of 99.1% of participants (438/442) had antibiotics before their surgical procedures, according to their hospital policy. More than half of the procedures (51.8%, 229/442) were performed out of normal daytime (07:00–17:00) working hours.

There were 161 (36.4%) open fractures that required IM nailings across the 2 study sites. Most of the open fractures were Gustilo–Anderson (GA) type I injuries (70.2%, 113/161) (see Appendix 1, Supplemental Digital Content, http://links.lww.com/QAI/B665). There were 95 gunshot wound (GSW) fractures, and most of these resulted in GA type I injuries (96.8%, 92/95); 92 of 161 (57.1%) open fractures were due to low-velocity GSW fractures. After open injuries, 97.5% (157/161) of participants received antibiotics within 24 hours of their injury, and all participants were given antibiotics before their surgical procedure according to hospital guidelines. A high proportion of participants with open fractures underwent IM nailing as a single procedure, without an initial washout or application of external fixator (88.8%, 143/161). At both study sites, all patients with low-velocity GSW fractures had their bullet entry and exit wounds left to heal by secondary intention and were given 24 hours of intravenous antibiotics perioperatively.

Of the 400 participants recruited to the study, 42 (10.5%, 47 IM nailings) of them were lost to follow-up before reaching a study outcome and were excluded from primary analysis. None of the 42 participants had developed a delayed union, a nonunion, a DSI, or an SSI before being lost to follow-up. Four participants (4 IM nailings) who were lost to follow-up were HIV-positive.

The population for final analysis, therefore, included 358 of the 400 (89.5%) participants, who underwent 395 IM nailings, all of whom were followed up for a minimum of 12 months. Seventy-one of the 358 (19.8%) participants were HIV-positive [83/395 (21%) IM nailings ].

### Radiographic Classification of Fracture Union

For the primary outcome of union, there were discrepant RUST scores in only 11 of the 395 (2.8%) radiographs (interobserver agreement 97.7% and Kappa = 0.92). At 9 months, there were 2 discrepancies in 69 (2.9%) radiographs taken to determine the secondary outcome of nonunion (interobserver agreement 97.1%, Kappa = 0.94).

### Primary Outcome: Delayed Fracture Union

Overall, 17.5% (69/395) of fractures had delayed union at 6 months (Table 3). No participant had more than one fracture that developed delayed union. Women made up 23.7% (85/358) of the study population and were significantly less likely on univariable analysis to have delayed union compared with men (OR: 0.38, 95% CI: 0.16 to 0.78; *P* = 0.014). However, on multivariable analysis, although delayed union was still less likely in women, this difference was not statistically significant (OR: 0.41, 95% CI: 0.17 to 1.01; *P* = 0.053).

**TABLE 3.** Associations Between HIV Status and Fracture Delay or Nonunion

	HIV-Positive (N = 83), n (%)	HIV-Negative (N = 312), n (%)	Univariable Odds Ratio (95% CI)	Multivariable Odds Ratio (95% CI)
Delayed union at 6 mo	12 (14.5)	57 (18.3)	0.76 (0.369 to 1.44)	1.06 (0.50 to 2.22)*
Nonunion at 9 mo	1 (1.2)	22 (7.1)	0.16 (0.01 to 0.78)	0.17 (0.01 to 0.92)†

* Adjusted for age, sex, smoking status, open fracture status, DSI, vitamin D level, and fracture site.

† Adjusted for age, sex, smoking status, open fracture status, hemoglobin level, and vitamin D level.

A total of 14.5% (12/83) of fractures in HIV-positive participants developed delayed union compared with 18.3% (57/312) fractures in the HIV-negative cohort. On both univariable (OR: 0.76, 95% CI: 0.37 to 1.44; *P* = 0.417) and multivariable (OR: 1.06, 95% CI: 0.50 to 2.22; *P* = 0.869) logistic regression models, there was no statistically significant difference in delayed union between HIV-positive and HIV-negative participants.

A greater number of ART-naïve participants with HIV developed delayed union (23.3%, 7/30) compared with those who were taking ART (12.2%, 5/41; *P* = 0.227). Baseline CD4 cell counts in participants with HIV who developed delayed union were similar to those in participants with HIV who did not develop delayed union (460 cell/mm^3^, IQR: 366–477 vs. 413 cell/mm^3^, IQR 295–673; *P* = 0.400, respectively). The median baseline HIV viral load in participants with HIV who developed delayed union was significantly higher than in participants with HIV whose fractures healed (3.02 log_10_ copies/mL, IQR: 0.98–4.76 log_10_ copies/mL vs. 2.13 log_10_ copies/mL, IQR: 1.30–4.40 log_10_ copies/mL; *P* = 0.001). This measurement included participants with detectable and undetectable viral loads.

Open fractures resulted in 28.5% (39/137) of all delayed bone union cases compared with 11.6% (30/258) of closed injuries. Open fractures were associated with more than 3 times greater odds of developing delayed bone union compared with closed fractures in both univariable (OR: 3.02, 95% CI: 1.78 to 5.18; *P*= 0.001) and multivariable (OR: 3.13, 95% CI: 1.74 to 5.63; *P* = 0.001) logistic models. The proportion of open fractures that developed delayed union was similar in HIV-positive (27.3%, 6/22) and HIV-negative (28.7%, 33/115) participants (univariable logistic model OR: 0.93, 95% CI: 0.31 to 2.49; *P* = 0.89).

### Secondary Outcome: Fracture Nonunion

At 9 months, 5.8% (23/395) of fractures had developed a nonunion (Table 3). A higher percentage of fractures in HIV-negative participants (7.1%, 22/312) experienced nonunion compared with that in HIV-positive participants (1.2%, 1/83). These associations were statistically significant in both univariable (OR: 0.16, 95% CI: 0.01 to 0.78) and multivariable (OR: 0.17, 95% CI: 0.01 to 0.92) logistic regression models.

Open fractures resulted in 65.2% (15/23) of all nonunions. A total of 10.9% (15/137) of all open fractures developed a nonunion compared with 3.1% (8/258) of closed fractures that developed a nonunion. On multivariable analysis, nonunion was nearly 3 times more likely after an open fracture (OR: 2.96, 95% CI: 1.16 to 8.07; *P* = 0.026). The proportion of participants who developed nonunion after an open fracture was lower in participants with HIV compared with that in those without HIV (0%, 0/22 vs. 13.0%, 15/115).

### Secondary Outcome: Infection

A total of 5.3% (21/395) of cases developed DSIs (Table 4); 8.4% (7/83) of fractures in participants with HIV developed DSI compared with 4.5% (14/312) of fractures that developed DSI in participants without HIV. HIV status was not significantly associated with DSI after IM nailing in the univariable model (OR: 1.96, 95% CI: 0.72 to 4.89; *P* = 0.161) or in the multivariable model (OR: 2.59, 95% CI: 0.86 to 7.80; *P* = 0.090). Three fractures in participants who developed DSI went on to delayed union; 2 of these were in HIV-positive participants (2/7, 28.6% vs. 1/14, 7.1%). No HIV-positive participants with HIV and one participant without HIV developed a nonunion after a DSI (0/7, 0% vs. 1/14, 7.1%).

**TABLE 4.** Outcomes of Infection, Stratified According to HIV Status

	HIV-Positive (N = 83), n (%)	HIV-Negative (N = 312), n (%)
DSI, n (%)	7 (8.4)	14 (4.5)
Superficial surgical site infection, n (%)	1 (1.2)	5 (1.6)
Late infection, n (%)	5 (6.0)	2 (0.6)

Only 1.5% (6/395) of fractures developed an SSI (Table 4); owing to these low numbers, univariable and multivariable logistic regression analyses were not undertaken. The proportion of fractures that developed SSIs in participants with HIV was 1.2% (1/83) compared with 1.6% (5/312) in participants without HIV. Two fractures that developed SSIs subsequently went on to nonunion (both HIV-negative), and no cases went onto delayed union.

Late implant infection developed in 1.8% (7/395) of all fractures (Table 4). Of these late infections, 1.3% (5/395) were in fractures in participants with HIV, compared with 0.5% (2/395) in participants without HIV. The proportion of fractures that developed late infection in participants with HIV was 6.0% (5/83) compared with 0.6% (2/312) in participants without HIV. Two late infections developed delayed union, both of which were in HIV-positive individuals (2/5, 40.0% vs. 0/2, 0%), and one late infection in an HIV-negative participant developed nonunion.

## DISCUSSION

To the authors' knowledge, this is the first large prospective study to assess the association between HIV infection and bone healing after a fracture. Previous small studies (fewer than 7 participants) reported delayed union in HIV-positive individuals.^16,27^ This study showed that HIV-positive status was not associated with the development of delayed bone healing after an IM nailing of the tibia or femur among trauma patients in the Western Cape, South Africa. In fact, there were lower odds of fracture nonunion in HIV-positive participants compared with that in HIV-negative participants. Previous studies had suggested nonunion in 0%–11% of fractures in HIV-positive individuals after surgical fixation of a fracture.^14^

Antiretroviral therapy regimen, CD4 count, and viral load at baseline were also not associated with a significant risk of delayed union in the study population of HIV-positive participants. However, a greater number of ART-naïve participants and more of those with a higher viral load developed delayed union, and a much larger, appropriately powered, study is required to investigate this further.

HIV was not associated with the development of delayed union or nonunion in open fractures. There is little evidence surrounding the risk of delayed union and nonunion after an open fracture in HIV-positive individuals in the current literature, but nonunion rates between 10% and 43% have been reported in a small number of studies.^14^

Contrary to previous research, this study demonstrated that HIV status does not seem to affect the clinical outcome of fracture healing and may potentially lower the risk of nonunion. This could be explained by a number of factors, including direct and indirect immunological effects of HIV on bone,^28,29^ and/or characteristics of the study population. However, this remains speculative because the study did not investigate the mechanisms of bone healing. It is also acknowledged that this study focused solely on individuals who underwent IM nailing of a lower limb long bone fracture. Therefore, our results may not translate to fractures at different sites of the musculoskeletal system, fractures treated by other methods of fixation, or fractures managed nonoperatively.

Infection rates in the study population as a whole were similar to those published in the literature.^30^ There was a low number of SSIs overall (1.5%, 6/395) in the study population, so it was difficult to make comparisons between HIV-positive and HIV-negative populations. The proportion of DSIs was higher in HIV-positive participants (8.4%, 7/83 vs. 4.5%, 14/312); however, this difference was not significant on multivariable analysis (OR: 2.59, 95% CI: 0.86 to 7.80; *P* = 0.090). If DSIs and SSIs were combined to give a rate of “early implant infection,” participants with HIV had an early implant infection rate of 9.6% (8/83) compared with 6.1% (19/312) in the population of participants without HIV. Late implant infection occurred in a higher proportion of HIV-positive than in HIV-negative participants (6.0%, 5/83 vs. 0.6%, 2/312). Overall, this suggests that infection was slightly more common in those who are HIV-positive, but the relatively low numbers make it difficult to draw substantial conclusions.

When assessing the role of infection and the development of delayed union and nonunion, participants with HIV who developed an SSI, DSI, or late infection were more likely to go onto delayed union (7/13, 53.8% vs. 1/21, 4.8%) but not nonunion (0/13, 0% vs. 4/21, 19.0%). However, the prevalence of infection overall was too low to make definitive conclusions, and much larger studies would be needed to investigate this further.

Of the 75 participants with HIV enrolled in the study, 56% (42/75) were taking ART on enrollment. Ideally, to determine the effect of HIV infection alone on fracture healing, the study should have included only those participants who were not on ART. However, ethical restrictions and time limitations made this impossible. Another limitation was the use of the RUST score to determine the primary outcome of delayed union. This scoring system has not been validated for use in the femur. However, it is the best tool available to determine bone union without the need for additional investigations.

This study demonstrated that HIV was not shown to be associated with the risk of developing delayed bone healing after an IM nailing of the tibia or femur in the study population. There was a strong trend toward lower odds of fracture nonunion in HIV-positive participants compared with that in HIV-negative participants. In conclusion, the evidence from this study suggests that fracture surgery in individuals with HIV is safe and as least as effective in HIV-positive as HIV-negative patients, with no increased risk of delayed union or nonunion. The results show that HIV status should not influence the decision to operate or use internal fixation in these patients.
